# Preoperative risk factors predict perioperative allogenic blood transfusion in patients undergoing primary lung cancer resections: a retrospective cohort study from a high-volume thoracic surgery center

**DOI:** 10.1186/s12893-023-01924-9

**Published:** 2023-02-27

**Authors:** Mircea Gabriel Stoleriu, Michael Gerckens, Julia Zimmermann, Johannes Schön, Fuad Damirov, Nicole Samm, Julia Kovács, Elvira Stacher-Priehse, Christina Kellerer, Rudolf A. Jörres, Teresa Kauke, Christian Ketscher, Uwe Grützner, Rudolf Hatz

**Affiliations:** 1 Division of Thoracic Surgery Munich, Hospital of Ludwig-Maximilians-University Munich (LMU), Marchioninistr. 15, 81377 Munich, Germany; 2 Department of Thoracic Surgery, Asklepios Pulmonary Hospital, Robert-Koch-Allee 2, 82131 Gauting, Germany; 3 Comprehensive Pneumology Center (CPC), Member of the German Lung Research Center, Helmholtz Zentrum Muenchen, Institute for Lung Biology and Disease, 81377 Munich, Germany; 4 Department of Internal Medicine V, Ludwig-Maximilians-University Munich (LMU), Marchioninistr. 15, 81377 Munich, Germany; 5 Department of Pathology, Asklepios Pulmonary Hospital, Robert-Koch-Allee 2, 82131 Gauting, Germany; 6 Institute and Outpatient Clinic for Occupational, Social and Environmental Medicine, Hospital of Ludwig-Maximilians-University Munich (LMU), Ziemssenstraße 1, 80336 Munich, Germany; 7 School of Medicine, Institute of General Practice and Health Services Research, Technical University of Munich, Orleansstr. 47, 81667 Munich, Germany; 8 Asklepios Lung Clinic Munich-GautingDivision of Thoracic Surgery Munich, Hospital of Ludwig-Maximilians-University Munich (LMU) and Asklepios Pulmonary Hospital, Robert-Koch-Allee 2, 82131 Gauting, Germany

**Keywords:** Lung cancer, Thoracic surgery, Perioperative blood transfusion, Rhesus factor

## Abstract

**Background:**

Our study aimed to identify preoperative predictors for perioperative allogenic blood transfusion (ABT) in patients undergoing major lung cancer resections in order to improve the perioperative management of patients at risk for ABT.

**Methods:**

Patients admitted between 2014 and 2016 in a high-volume thoracic surgery clinic were retrospectively evaluated in a cohort study based on a control group without ABT and the ABT group requiring packed red blood cell units within 15 days postoperatively until discharge. The association of ABT with clinically established parameters (sex, preoperative anemia, liver and coagulation function, blood groups, multilobar resections) was analyzed by contingency tables, receiver operating characteristics (ROC) and logistic regression analysis, taking into account potential covariates.

**Results:**

60 out of 529 patients (11.3%) required ABT. N1 and non-T1 tumors, thoracotomy approach, multilobar resections, thoracic wall resections and Rhesus negativity were more frequent in the ABT group. In multivariable analyses, female sex, preoperative anemia, multilobar resections, as well as serum alanine-aminotransferase levels, thrombocyte counts and Rhesus negativity were identified as independent predictors of ABT, being associated with OR (95% Confidence interval, p-value) of 2.44 (1.23–4.88, p = 0.0112), 18.16 (8.73–37.78, p < 0.0001), 5.79 (2.50–13.38, p < 0.0001), 3.98 (1.73–9.16, p = 0.0012), 2.04 (1.04–4.02, p = 0.0390) and 2.84 (1.23–6.59, p = 0.0150), respectively.

**Conclusions:**

In patients undergoing major lung cancer resections, multiple independent risk factors for perioperative ABT apart from preoperative anemia and multilobar resections were identified. Assessment of these predictors might help to identify high risk patients preoperatively and to improve the strategies that reduce perioperative ABT.

**Supplementary Information:**

The online version contains supplementary material available at 10.1186/s12893-023-01924-9.

## Background

Perioperative allogenic blood transfusion (ABT) is frequent in lung cancer patients and associated with high morbidity. While postoperative bleeding occurs rarely in primary lung tumor surgery (1.3–2.1%) [1, 2], ABT occurs in 9–55.4% of patients [3, 4]. ABT carries a risk for complications, negatively impacts prognosis [5] and is limited by the availability of red blood cell (RBC) units. Therefore, preoperative identification of risk factors for perioperative ABT in lung cancer surgery might help to reduce ABT use and improve surgery outcomes [6].

Moreover, preoperative blood management strategies to reduce the need for ABT rely on the identification of patients at risk. Among the well-understood predictors of perioperative ABT is preoperative anemia which is found in 50–60% of lung cancer patients [7]; these patients require ABTs frequently (25.7–43%) [3, 8, 9].

Predictors other than preoperative anemia might further help to identify patients at risk for ABT, but are currently less well understood. Here, clinical chemistry parameters, blood counts and blood group systems including Rhesus factor, as well as tumor size and histology might play a role [10]. A panel of risk factors could help to significantly reduce ABT orderings for a low-risk collective or to improve perioperative anemia management in high-risk individuals using targeted preemptive measures such as erythropoiesis stimulation or autotransfusion protocols.

Based on this, the aim of the present study was to evaluate a broad panel of preoperative, easily available clinical parameters in relation to perioperative ABT in patients undergoing primary lung cancer resections in a high-volume thoracic surgery clinic.

## Methods

### Study population

This monocentric retrospective cohort study was performed in accordance with the Declaration of Helsinki, after approval by the Ethics Committee of the Ludwig-Maximilians-University Munich (LMU), Germany (#21-0386). It was conducted at the Division for Thoracic Surgery Munich in the Asklepios Lung Clinic Munich-Gauting, Germany. The study analyzed the occurrence of the outcome (ABT) in the exposed patients (i.e. experiencing lung cancer resections) reported according to the STROBE recommendations.

### Exposure

**Fig. 1:**
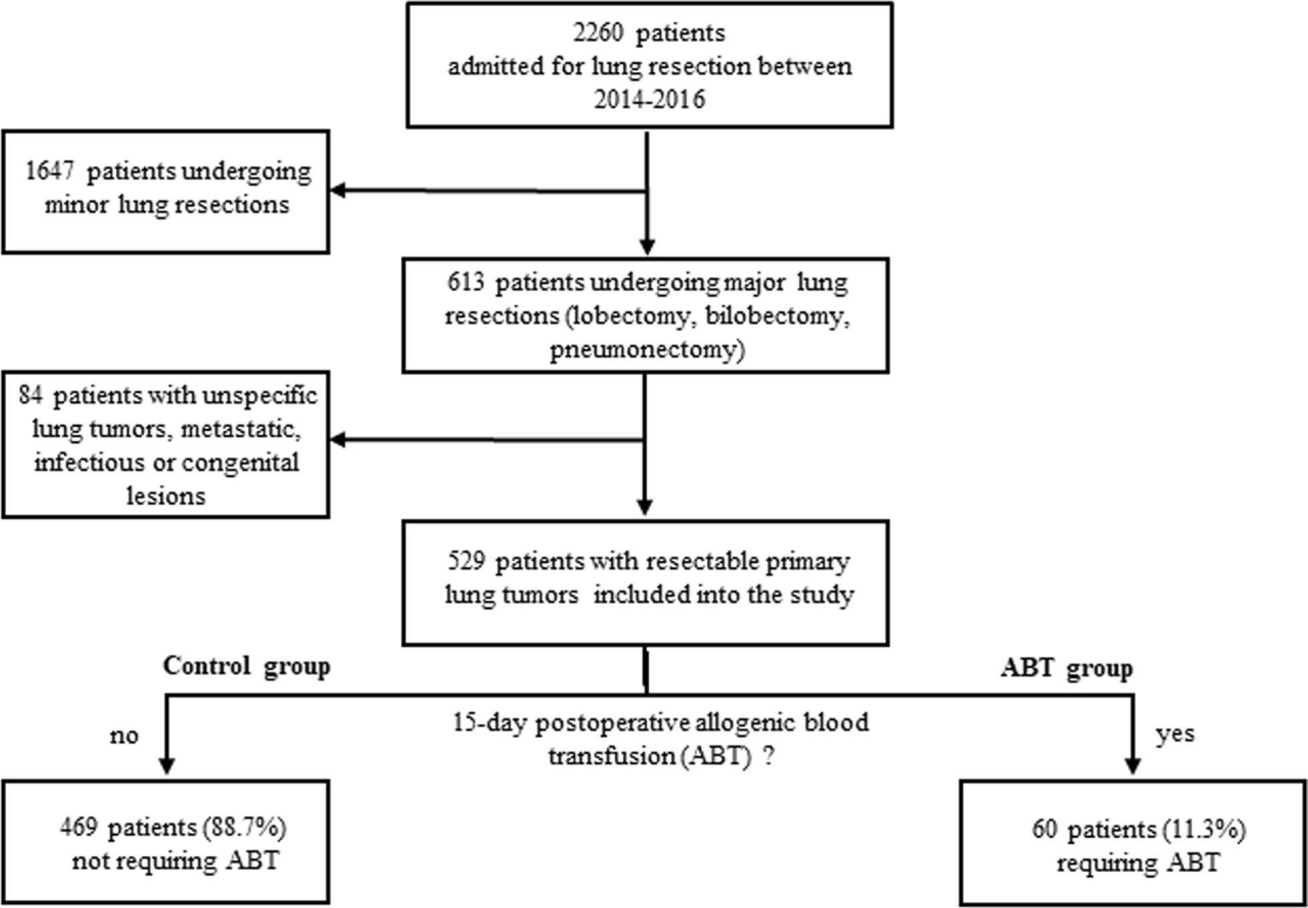
Study flow chart illustrating patient enrollment at study entry. Of 2260 patients undergoing thoracic surgery, 1647 (72.9%) patients underwent minor lung resections or non-pulmonary resections. Eighty-four (3.7%) patients experiencing intrathoracic sarcoma, mesothelioma, unclassifiable tumors, pulmonary metastatic lesions, and benign lung tumors, infectious or congenital processes were excluded from the study, thus 529 of 2260 patients (23.4%) with primary resectable lung tumors were included. Based on the need for ABT, patients were categorized into two groups: Control group (469 patients, 88.7%) without need for postoperative blood administration, and ABT group (60 patients, 11.3%) with postoperative ABT requirements

All patients with resectable malignant primary lung tumors treated by major surgical resections (lobectomy, bilobectomy or pneumonectomy) between January 2014 and December 2016 were included. Patients undergoing minor lung resections (for malignant or non-malignant lung lesions, n = 1647) or experiencing unspecific, metastatic, infectious or congenital lung lesions (n = 84) admitted for major pulmonary resections were excluded (Fig. 1).

The ABT group comprised patients with in-hospital allogenic blood transfusion events until discharge and within 15 days postoperatively, the control group patients without ABT until discharge and within 15 days postoperatively. This time period was chosen as being the median in-hospital stay time for the whole patient population.

### Outcome

Regarding blood transfusion, only patients with RBCs transfusions were considered (ABT group). Fresh frozen plasma products (FFPs) and platelet pack requirements were not investigated due to the very low number of observed events. Accordingly, patients who received only FFPs or platelet packs were included into the control group. Patients requiring both RBCs and FFPs or RBCs and platelet packs were included in the ABT group.

### Data assessments/sources

Clinical data were collected from patients’ files and the database of the Munich Cancer Registry. Primary lung tumors were categorized according to the 7th edition of the TNM staging system, and histopathological analysis was performed according to the World Health Organization Classification of lung tumors [11].

Information on blood group systems and perioperative ABT requirements were obtained from the internal Transfusion Medicine and Blood Bank Facility. Clinical data included demographics (age, sex, BMI, comorbidities), neoadjuvant chemotherapy, preoperative anticoagulants, preoperative standard laboratory and lung function parameters, TNM classification, WHO histology of primary tumor, description of the surgical approach (video-assisted thoracoscopic surgery (VATS)/conversion to open surgery/primarily open surgery, surgical time, blood loss), postoperative stay, as well as data on ABO and Rhesus blood group systems. Oral anticoagulant and antiaggregant medications except for acetylsalicylic acid were routinely paused or bridged with heparin products to ensure normalized coagulation status during surgery. The following laboratory parameters were collected on the admission day (usually 1–3 days before surgery) according to the in-house standard: blood counts, international normalized ratio (INR), partial thromboplastin time (PTT), fibrinogen, C-reactive protein (CRP), creatinine, urea, estimated glomerular filtration rate (eGFR), alanine-aminotransferase (ALAT), aspartate-aminotransferase (ASAT), gamma-glutamyl transferase (GGT), lactate dehydrogenase (LDH), carcinoembryonic antigen (CEA), cytokeratin-fragment 19 (CYFRA 21-1), and neuron-specific enolase (NSE). Prior to surgery, the anticoagulation status was routinely checked and a careful bridging strategy with heparin or low molecular weight heparin was used.

Preoperative anemia was defined according to the WHO classification using sex-specific cut-off values (female < 12 g/dL, male < 13 g/dL).

ABT was performed in accordance with the Guidelines for Therapy with blood components and Plasma derivatives published by the German Federal Medical Association in 2014 (https://www.bundesaerztekammer.de/fileadmin/user_upload/downloads/QLL_Haemotherapie_2014.pdf). Accordingly, patients with serum hemoglobin levels between 6 and 8 g/dL with a medical cardiovascular or cerebrovascular history, hypoxia or chronic anemia postoperatively were subjected to ABT. Serum hemoglobin levels < 6 g/dL were considered as absolute indication for ABT. During surgery, antifibrinolytic and blood product usage including RBCs, FFPs and platelet packs was indicated based on the anesthesiologist’s and surgeon’s assessment in the case of objectively increased bleeding and blood loos.

The present analysis was explicitly based on variables that could be argued as clinically meaningful, such as sex, preoperative anemia, multilobar resection, liver function and blood groups including Rhesus factor. They were incorporated in the multivariable analysis, while taking into account further variables that could be considered as clinically relevant confounders, such as BMI, comorbidities, neoadjuvant chemotherapy, coagulation function and anticoagulant medication, preoperative chest surgery and surgical duration.

### Data analysis

Continuous variables are presented as median values and quartiles. Binary variables were analyzed by contingency tables using the chi-square test statistics and Fisher’s exact test. Laboratory findings were compared between groups using the Mann–Whitney U-test. Multivariable analysis was performed by binary logistic regression analysis. To evaluate the robustness of predictors in view of their mutual correlation, as well as the consistency of the results, we relied on the approach of including all variables, which was only supplemented by stepwise forward and backward selection. Odds ratios (OR) with 95% confidence intervals (CI) were used to assess the occurrence of the outcome in the exposed patients. To assess optimal cut-off values for laboratory parameters, receiver-operator characteristics (ROC) analysis and the Youden criterion were used. To check the findings on the post hoc analysis stratified according to anemia, the technique of decision trees using the exhaustive CHAID method was applied to the total group. We used tenfold cross-validation and additional checks by the alternative estimation procedures CRT and QUEST to deal with the potential problems of overfitting and statistical instability. All analyses were performed after excluding the missing data (found to be under 10% for selected variables), using the software Prism (Version 8.0, GraphPad, San Diego, CA) and SPPS (Version 26, IBM, Armonk, New York, USA). Results with type I error p < 0.05 were considered as significant.

## Results

### Study population

Of 2260 patients admitted for thoracic surgery, 529 patients fulfilled the inclusion criteria (233 female (44%), 296 (56%) male patients; median [quartiles] age of 67 [59; 73] years). Overall, 469 patients (control group, 88.7%) recovered without ABT use, whereas 60 patients (ABT group, 11.3%) required at least one, in median 2 [2; 4] RBCs perioperatively. Two patients received only FFPs, seven patients required both RBCs and FFPs and two patients required both RBCs and platelet packs. The maximum number of RBCs per patient was 10 in 2 patients. Of 60 patients, 27 (45%) required ABT intraoperatively, 30 (50%) on the surgery day, and 18 (30%) on the first day post-surgery. Overall, 47 patients (78%) received ABT in the first 3 days after surgery, 12 patients (20%) within 3 and 15 days post-surgery. The selection process and groups of patients are illustrated in Fig. 1.

### Standard laboratory parameters and ABT

Laboratory parameters of the two groups prior to surgery are illustrated in Table 1.

**Table 1:** Preoperative laboratory tests in patients undergoing major surgical resections classified by intra- and postoperative ABT requirements

Parameters preoperatively (median, quartiles [1st; 3rd])	Control group (no ABT) n = 469	ABT group n = 60	p value
Blood counts			
Leukocytes (/nL)	7.80 [6.45; 9.30]	8.65 [6.90; 10.33]	0.0736
Erythrocytes (/pL)	4.60 [4.30; 4.90]	3.90 [3.50; 4.20]	< 0.0001
Hemoglobin (g/dL)	14.0 [13.0; 14.9]	10.9 [10.3; 12.3]	< 0.0001
Hematocrit (%)	0.41 [0.39; 0.44]	0.34 [0.31; 0.37]	< 0.0001
MCV (fL)	90.0 [87.0; 93.0]	88.0 [83.3; 92.0]	0.0226
MCH (pg/cell)	30.3 [29.2; 31.4]	28.9 [27.5; 31.0]	< 0.0001
MCHC (g/dL)	33.8 [33.0; 34.4]	32.8 [32.1; 33.4]	< 0.0001
Thrombocytes (/nL)	269.0 [223.0; 313.5]	324.5 [236.3; 445.3]	< 0.0001
Blood coagulation			
INR	1.02 [0.97; 1.07]	1.07 [1.02; 1.14]	< 0.0001
PTT (s)	30.0 [28.0; 32.0]	30.05 [28.00; 33.10]	0.3096
Fibrinogen (mg/dL)	295.5 [250.0; 373.0]	427.0 [294.0; 526.0]	< 0.0001
Clinical chemistry			
CRP (mg/L)	3.30 [1.70; 8.80]	14.50 [4.13; 64.40]	< 0.0001
Creatinine (ng/mL)	1.0 [0.80; 1.10]	1.0 [0.80; 1.10]	0.3744
Urea (mg/dL)	31.0 [24.0; 38.0]	31.5 [24.3; 43.8]	0.2857
eGFR (mL/min) > 60 (n, %)	376/469 (80.2%)	43/60 (71.7%)	0.1264
ALAT (IU/L)	27.0 [21.5; 37.0]	23.0 [17.0; 32.0]	0.0027
ASAT (IU/L)	20.0 [16.0; 25.0]	20.0 [16.3; 25.8]	0.8248
GGT (IU/L)	33.0 [25.0; 50.0]	37.5 [27.3; 74.8]	0.0845
LDH (IU/L)	193.0 [170.8; 222.0]	199.5 [163.0; 252.0]	0.4550
Serum tumor markers			
CEA (ng/mL)	3.20 [1.88; 5.83]	3.00 [1.98; 7.30]	0.7046
CYFRA 21-1 (ng/mL)	1.70 [1.20; 2.70]	2.35 [1.40; 4.58]	0.0026
NSE (ng/mL)	17.35 [14.60; 20.83]	17.40 [14.65; 23.63]	0.7439

*ABT* allogenic blood transfusion; *INR* international normalized ratio; *MCV* mean corpuscular volume; *MCH* mean corpuscular hemoglobin; *MCHC* mean corpuscular hemoglobin concentration; *PTT* partial thromboplastin time; *CRP* C-reactive protein; *eGFR* estimated glomerular filtration rate; *ALAT* alanine-aminotransferase; *ASAT* aspartat-aminotransferase; *GGT* gamma-glutamyl transferase; *LDH* lactate dehydrogenase; *CEA* carcinoembryonic antigen; *CYFRA 21-1* cytokeratin-fragment 19; *NSE* neuron-specific enolase

The comparison revealed decreased erythrocyte numbers (p < 0.0001), mean corpuscular volume (MCV, p = 0.0226), hemoglobin (p < 0.0001) and ALAT (p = 0.0027) in the ABT compared to the control group. Conversely, thrombocytes, fibrinogen, CRP (p < 0.0001 each), and CYFRA 21-1 (p = 0.0026) were increased in the ABT group.

### Tumor characteristics, surgical approach and ABT

The relationship between ABT groups and tumor features is shown in Table 2.

**Table 2:** Demographics of patients undergoing surgical resection of primary lung tumors classified by ABT requirements

Patient demographics at study entry	Control group (no ABT) n = 469	ABT group n = 60	p value
Age (median, quartiles [1st; 3rd]) years	67.03 [59.3; 73.2]	66.7 [60.3; 72.3]	0.8436
Sex (n, %)			
Female	202/469 (43.1%)	31/60 (51.7%)	0.2066
Male	267/469 (56.9%)	29/60 (48.3%)	
BMI (median, quartiles [1st; 3rd])	26.1 [23.4; 29.4]	24.6 [21.1; 27.6]	0.0066
BMI < 18.5 kg/m^2^ (n, %)	14/451 (3.1%)	5/59 (8.5%)	0.0566
BMI > 30 kg/m^2^ (n, %)	104/451 (23.1%)	8/59 (13.6%)	0.0974
Comorbidities (n, %)			
Respiratory	186/467 (39.8%)	29/59 (49.2%)	0.1698
Cardiovascular	149/467 (31.9%)	19/59 (32.2%)	0.9632
Renal	28/467 (6.0%)	7/59 (11.9%)	0.0967
Liver	16/467 (3.4%)	2/59 (3.4%)	1.0
Neurological	58/467 (12.4%)	8/59 (13.6%)	0.8034
Diabetes mellitus	50/467 (10.7%)	5/59 (8.5%)	0.5975
Non-pulmonary malignancies	69/467 (14.8%)	13/59 (22.0%)	0.1476
Coagulation disorders	3/467 (0.6%)	0/59 (0.0%)	1.0
No. of comorbidities > 1	167/469 (35.6%)	26/60 (43.3%)	0.2418
Previous treatments (n, %)			
Previous thoracic surgery	18/458 (3.9%)	1/57 (1.8%)	0.7097
Neoadjuvant chemotherapy	24/467 (5.1%)	11/59 (18.6%)	0.0007
Preoperative anticoagulation	179/467 (38.3%)	25/59 (42.4%)	0.5481
Lung function parameters (median, quartiles [1st; 3rd])			
VC (predicted, %)	0.94 [0.83–1.04]	0.86 [0.73–0.96]	0.0013
FEV_1_ (predicted, %)	0.77 [0.69–0.84]	0.76 [0.68–0.86]	0.9262
DLCO (predicted, %)	0.73 [0.60–0.84]	0.55 [0.47–0.74]	< 0.0001
Tumor size (median, quartiles [1st; 3rd]) cm	3.10 [2.10–4.50]	4.50 [2.60–6.80]	0.0011
> 3 cm (n, %)	252/469 (53.7%)	44/60 (73.3%)	0.0039
Tumor side (n, %)			
Left	204/469 (43.5%)	29/60 (48.3%)	0.4774
Right	265/469 (56.5%)	31/60 (51.7%)	
Tumor localization (n, %)			
Left upper lobe	108/469 (23.0%)	14/60 (23.3%)	0.7197
Left lower lobe	73/469 (15.6%)	8/60 (13.3%)	0.7197
Right upper lobe	134/469 (28.6%)	15/60 (25.0%)	0.4884
Middle lobe	42/469 (9.0%)	5/60 (8.3%)	0.5859
Right lower lobe	88/469 (18.8%)	9/60(15.0%)	0.8921
Histological features of primary tumor (WHO 2015) (n, %)			
Non-small cell lung cancer	465/469 (99.1%)	60/60 (100%)	1.0
Adenocarcinoma	260/469 (55.4%)	22/60 (36.7%)	0.0060
G1 lepidic	22/469 (4.7%)	0/60 (0%)	0.2350
G2 acinar/papilar	153/469 (32.6%)	11/60 (18.3%)	0.4193
G3 micropapilar/solide	81/469 (17.3%)	9/60 (15.0%)	0.3459
Undifferentiated	4/469 (0.9%)	2/60 (3.3%)	0.0720
Squamous-cell carcinoma	122/469 (26.0%)	27/60 (45.0%)	0.0021
Keratinized	57/469 (12.2%)	16/60 (26.7%)	0.2150
Non-keratinized	60/469 (12.8%)	8/60 (13.3%)	
Basaloid	2/469 (0.4%)	1/60 (1.7%)	
Carcinoid	53/469 (11.3%)	2/60 (3.3%)	0.0569
Typical	47/469 (10.0%)	2/60 (3.3%)	
Atypical	6/469 (1.3%)	0/60 (0%)	
Large-cell	21/469 (4.5%)	3/60 (5.0%)	0.7456
Others	9/469 (1.9%)	6/60 (10.0%)	0.0037
Small cell lung cancer	4/469 (0.9%)	0/60 (0%)	1.0
TNM7 classification (n, %)			
T_0_	2/469 (0.4%)	1/60 (1.7%)	0.3036
T_1_	181/469 (38.6%)	8/60 (13.3%)	0.0001
T_2_	203/469 (43.3%)	24/60 (40.0%)	0.6285
T_3_	70/469 (14.9%)	24/60 (40.0%)	< 0.0001
T_4_	13/469 (2.8%)	3/60 (5.0%)	0.4100
Lymph node involvement (n, %)			
N_0_	319/469 (68.0%)	36/60 (60.0%)	0.2803
N_1_	64/469 (13.6%)	14/60 (23.3%)	0.0037
N_2_	84/469 (17.9%)	9/60 (15.00%)	0.6137
N_3_	2/469 (0.4%)	0/60 (0%)	1.0
Unknown	0/469 (0%)	1/60 (1.7%)	1.0

*ABT* allogenic blood transfusion; *BMI* body mass index; *VC* vital capacity; *FEV*_*1*_ forced expiratory volume in one second; *DLCO* diffusing capacity of the lung for carbon monoxide

Patients requiring ABT had more frequently tumors > 3 cm (p = 0.0039). Adenocarcinoma was the most frequent histopathological tumor type in the control group (p = 0.0060), while squamous-cell carcinoma was more frequent in the ABT group (p = 0.0021). T3 tumors (p < 0.0001) and N1 status (p = 0.0037) were more frequent in patients requiring ABT. Conversely, T1 tumors were more frequent in the control group (p = 0.0001).

Comorbidities and related factors (Table 2) were not significantly associated with ABT use. Specifically, BMI extreme categories, coagulation disorders, anticoagulation therapy, as well as previous chest surgery or chest re-exploration were not significantly associated with ABT use.

The characteristics of the surgical approach are illustrated in Table 3.

**Table 3:** Technical aspects of the tumor resection in primary lung cancer patients grouped by ABT requirements

Features of the surgical approach	Control group (no ABT) n = 469	ABT group n = 60	p value
Resection side (n, %)			
Left	204/469 (43.5%)	29/60 (48.3%)	0.4773
Right	265/469 (56.5%)	31/60 (51.7%)	
Surgical approach (n, %)			
Open (thoracotomy)	365/469 (77.8%)	57/60 (95.0%)	0.0018
Minimally invasive (VATS)	104/469 (22.2%)	3/60 (5.0%)	
Conversion to open	31/151 (20.5%)	2/3 (66.7%)	0.1162
Re-exploration/revision	7/467 (1.5%)	1/59 (1.7%)	0.9077
Resection extent (n, %)			
Lobectomy	416/469 (88.7%)	41/60 (68.3%)	< 0.0001
Multilobar—bilobectomy	19/469 (4.1%)	6/60 (10.0%)	0.0521
Pneumonectomy	34/469 (7.2%)	13/60 (21.7%)	0.0002
Topographical resection (n, %)			
Sleeve resection	49/469 (10.4%)	10/60 (16.7%)	0.1496
Thoracic wall	15/469 (3.2%)	8/60 (13.3%)	0.0021
Great vessels reconstruction	33/469 (7.0%)	5/60 (8.3%)	0.7891
Anaesthesia time (median, quartiles [1st; 3rd]) (minutes)	210 [140; 260]	238 [0.0; 289]	0.0976
Surgery time (median, quartiles [1st; 3rd]) (minutes)	160 [95; 205]	180 [0.0; 224]	0.1644
Intraoperative blood loss (median, quartiles [1st; 3rd]) (mL)	200 [200; 300]	300 [200; 675]	< 0.0001
Length of stay (median, quartiles [1st; 3rd]) (days)	15.0 [13.0; 19.0]	17.5 [15.0; 22.0]	0.0011

*ABT* allogenic blood transfusion; *VATS* video-assisted thoracoscopic surgery

A conversion to open surgery was needed in 33 out of 529 patients (6%) and was not significantly associated with ABT use (p = 0.1162). In this small subgroup of patients only two patients required perioperative ABT.

ABT was more frequent with open surgical procedures (p = 0.0018), and patients from the ABT group had more frequently (p < 0.0001) multilobar resections. In addition, thoracic wall resections were more frequent in ABT patients (p = 0.0021). From eight patients experiencing a chest re-exploration (1.5%), only one patient required ABT (p = 0.9077).

### Blood group systems and ABT

The frequency of the blood groups O, A, B and AB was 40.8%, 41.8%, 12.3% and 5.1%, respectively. Of 529 patients, 458 patients (86.6%) had Rhesus factor (Rh+, D phenotype), whereas 71 patients (13.4%) were characterized by the absence of Rhesus factor (Rh−, dd phenotype). ABT was significantly more frequent in Rhesus-negative patients (11.7% vs 26.7%, p = 0.0013, Table 4). C and E phenotypes were not significantly different between both groups.

**Table 4:** Characterization of blood groups systems in patients undergoing major surgical resections classified by ABT requirements

Characterization of blood groups systems	Control group (no ABT) n = 469	ABT group n = 60	p value
ABO (n, %)			
O	194/469 (41.4%)	22/60 (36.7%)	0.4857
A	190/469 (40.5%)	28/60 (46.7%)	0.3618
B	58/469 (12.4%)	7/60 (11.7%)	0.8764
AB	24/469 (5.1%)	3/60 (5.0%)	1.0
Rhesus/D phenotype (n, %)			
Positive (DD, Dd)	414/469 (88.3%)	44/60 (73.3%)	0.0013
Negative (dd)	55/469 (11.7%)	16/60 (26.7%)	
ABT			
≥ 1 RBCs (n, %)	0/469 (0%)	60/60 (100%)	
No. RBCs (median, quartiles [1st; 3rd])		2 [2; 4]	

*ABT* allogenic blood transfusion; *RBCs* red blood cells units

No association between ABO blood group system and tumor localization, histology, extent and TNM stage was observed.

### Logistic regression analysis of risk factors

Clinically meaningful and easily available parameters (sex, preoperative anemia, multilobar resection, liver and coagulation function, as well as Rhesus factor) were incorporated in the multivariable analysis to assess their independent predictive value. Compared to the univariate results, the following variables were confirmed as statistically significant (p < 0.05 each, Table 5): preoperative anemia, ALAT < 17.5 IU/L, thrombocytes > 293.5/nL, Rhesus factor negativity, multilobar resection based on the inclusion of all variables. When applying forward and backward selection, this was confirmed, demonstrating female sex, preoperative anemia, multilobar resection, ALAT, thrombocytes and Rhesus negativity as independent predictors of ABT. The corresponding odds ratios (OR) for ABT and their 95% confidence intervals were 2.44 (1.23–4.88, p = 0.0112), 18.16 (8.73–37.78, p < 0.0001), 5.79 (2.50–13.38, p < 0.0001), 3.98 (1.73–9.16, p = 0.0012), 2.04 (1.04–4.02, p = 0.0390) and 2.84 (1.23–6.59, p = 0.0150). The results of the logistic regression analysis are summarized in Table 5. An additional analysis by considering the intraoperative blood loss in the final regression model was summarized in Additional file 1: Table S1.

**Table 5:** Binary logistic regression model predicting postoperative ABT requirements in primary lung cancer patients undergoing major surgical resections

Covariates for postoperative ABT	Exp(B) [95% CI]	p value
Sex (female)	2.44 [1.23–4.88]	0.0112
Preoperative anemia	18.16 [8.73–37.78]	< 0.0001
Multilobar resection	5.79 [2.50–13.38]	< 0.0001
ALAT < 17.5 IU/L	3.98 [1.73–9.16]	0.0012
Thrombocytes > 293.5/nL	2.04 [1.04–4.02]	0.0390
Rh− (dd phenotype)	2.84 [1.23–6.59]	0.0150

Exp(B) = Odds ratio, 95% Confidence interval [lower bound − upper bound]

*ALAT* alanin-aminotransferase; *Rh*− Rhesus factor negativity

In female sex, postmenopausal age (> 50 years) was not significantly related to perioperative ABT. Despite being statistically significant in univariate comparisons, other variables (neoadjuvant chemotherapy, preoperative vital capacity (VC, % predicted) or diffusion lung capacity for carbon monoxide (DLCO, % predicted), surgical approach, thoracic wall resection, histology, tumor stage, hilar lymph node involvement, INR, serum levels of fibrinogen, CRP and CYFRA 21-1) were not significantly associated with perioperative ABT in multivariable analyses when adding them separately to the variables shown in Table 5. In the same way, the multivariable analysis was repeated by incorporating different potential confounders (neoadjuvant chemotherapy, BMI, preoperative chest surgery, surgical duration, perioperative anticoagulation management) into the regression model, with no statistical significance of these confounders. The multivariable analysis was also reproduced as sensitivity analysis when grouping patients receiving only FFPs into ABT group, with qualitatively unchanged results (Additional file 2: Table S2).

### Subgroup analysis stratified for anemia

Hemoglobin was the strongest predictor of ABT and anemia is well known as risk factor for ABT. Due to its strong influence, it may well be that the predictive value of other variables depends on the presence or absence of anemia. We thus investigated whether the remaining predictors played a different role in patients with anemia versus those without, to help in avoiding the unnecessary consideration of variables in one of the groups.

**Fig. 2:**
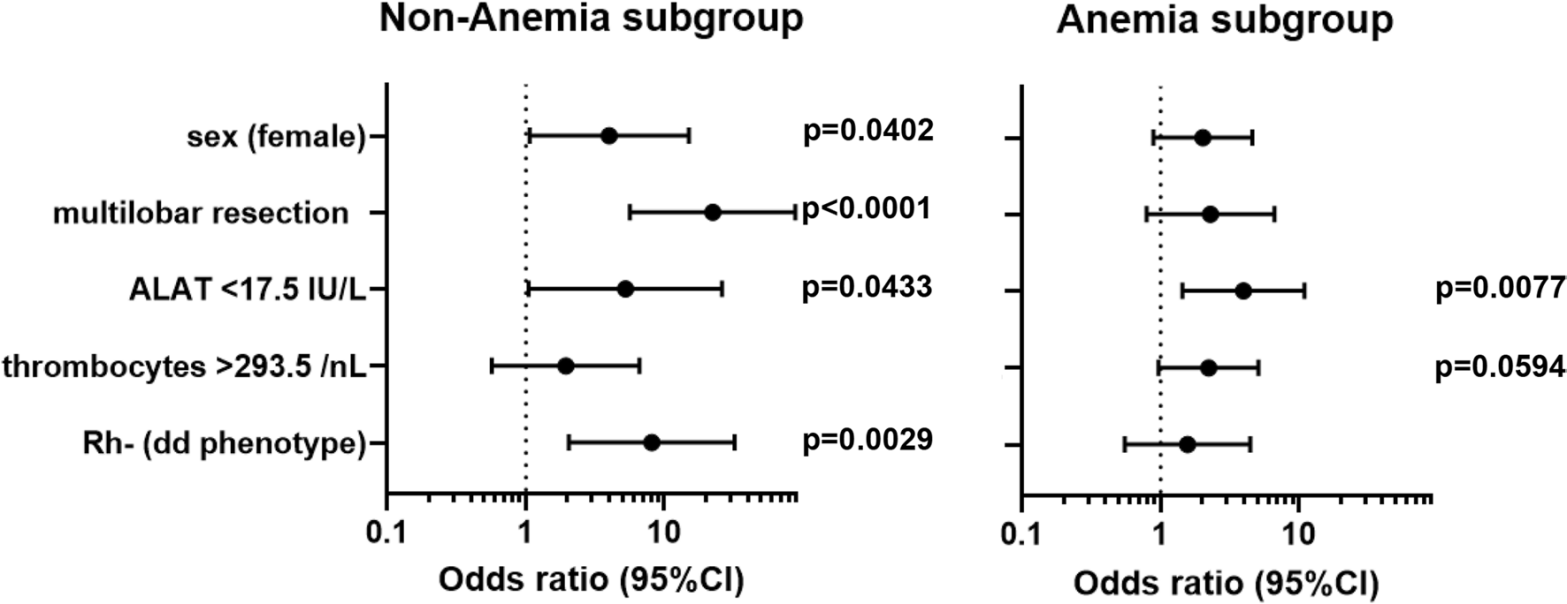
Forest plot illustrating the preoperative determinants of postoperative ABT categorized by sex-specific hemoglobin levels. In non-anemic patients, female sex, multilobar resection, Rhesus factor negativity and ALAT < 17.5 IU/L were associated with increased odds for postoperative ABT. In anemic patients, ALAT < 17.5 IU/L was associated with increased odds of postoperative ABT. Dots represent odds ratios, whiskers 95%-confidence intervals. P-values are also given

Therefore, the logistic regression analyses were repeated for the subgroups with and without anemia. This revealed that multilobar resection (OR (95% Confidence interval, p-value) of 22.36 (5.65–88.46, p < 0.0001)) and Rhesus negativity (OR (95% Confidence interval, p-value) of 8.11 (2.05–32.10, p = 0.0029)) were the main predictors for perioperative ABT in non-anemic patients, while low ALAT levels < 17.5 IU/L (OR (95% Confidence interval, p-value) of 3.98 (1.44–10.97, p = 0.0077)) played a role for ABT use in anemic patients (Fig. 2, Additional file 3: Fig. S1).

While Rhesus negativity was not a relevant predictor in patients with preoperative anemia (OR (95% Confidence interval, p-value) of 1.57 (0.55–4.45, p = 0.3978)), it was predictive in non-anemic patients (OR (95% Confidence interval, p-value) of 8.11 (2.05–32.10, p = 0.0029)). This result was consistently verified with three different estimation procedures for constructing a decision tree (Exhaustive CHAID, CRT, QUEST) suggesting a certain degree of robustness and supporting the result that the predictive value of variables decisively depended on the anemia status (Additional files 4, 5: Fig. S2, Fig. S3).

To determine whether the results depended on the inclusion of the Rhesus factor, we repeated the multivariable analyses after excluding this variable from the predictors. Preoperative anemia, female sex, multilobar resection, ALAT and thrombocytes remained as independent ABT predictors (OR (95% Confidence interval, p-value) of 18.22 (8.84–37.52, p < 0.0001), 2.38 (1.20–4.69, p = 0.0127), 5.36 (2.36–12.18, p < 0.0001), 4.08 (1.81–9.20, p = 0.0007), 2.13 (1.10–4.16, p = 0.0260)), thereby underlining the statistical robustness of our findings.

## Discussion

The aim of the study was to analyze a broad panel of preoperative, easily available and clinically meaningful parameters that could predict perioperative ABT use in resected lung cancer patients. For this purpose, a large cohort from a high-volume thoracic surgery clinic was studied. The proportion of perioperative ABT (11.3%) was in line with reported values. A wide range of perioperative ABT rates (9–55.4%) was reported in previous studies [3, 4]. These variations may probably reflect the multifactorial etiology of the anemia and the differences in the perioperative transfusion management in different hospitals. Our analysis included only RBCs orderings for ABT use, as the administrations of FFPs and platelet packs only were observed rarely. However, when categorizing FFPs and platelet packs into the ABT group, qualitatively similar results were reported. Thus, our results did not critically depend on these products.

The analysis comprised clinically established parameters (sex, preoperative anemia, blood groups, liver and coagulation function, TNM, resection extent) as well as potential confounders (neoadjuvant chemotherapy, BMI, preoperative chest surgery, surgical duration). We also included blood group systems which we considered not sufficiently studied with regard to their association with ABT.

The laboratory panel that we included is available in the majority of the Thoracic Surgery and Pneumology departments in Germany and routinely determined upon admission. This fact would enable an uncomplicated incorporation of these potential predictors into a routine ABT risk stratification.

The fact that we confirmed the correlation between preoperative anemia and postoperative ABT [12] was important as it underlined that our data were valid and thus probably allowed for further conclusions. The further parameters included decreased ALAT levels and lung function parameters (VC and DLCO), increased thrombocyte counts, INR, fibrinogen, GGT, CRP, CYFRA 21-1 levels, as well as Rhesus negativity, neoadjuvant chemotherapy, multilobar resections and tumor size > 3 cm (T2). These variables were found in univariate analyses to be associated with ABT use. In the multivariable analysis we confirmed female sex, preoperative anemia, multilobar resections, ALAT, thrombocytes, and Rhesus negativity as independent predictors of ABT. The association with these parameters was statistically robust across a variety of multivariable models considering all available confounders.

From a clinical point of view, anemia probably resulted from iron deficiency or anemia of chronic disease associated with chemotherapy, tumor inflammation and reduced hematopoiesis; a low ALAT level probably reflected an altered liver function associated with sarcopenia and frailty in chronically ill patients [13]; while an increased thrombocyte level was probably associated with systemic inflammation and tumor growth. These assumptions are in line with the increased CRP and fibrinogen levels as further factors associated with anemia of chronic disease, as well as tumor-associated systemic inflammation that is linked to poor survival in lung cancer patients [14]. To summarize, ABT is probably directly associated with an increased inflammatory status, altered coagulation and impaired liver function, all of which are clinically plausible associations demonstrated in previous studies [15–17]. These results were reflected in the significant association between elevated thrombocytes, fibrinogen and CRP levels as well as low ALAT levels on the one side, and preoperative anemia on the other side.

Our study addressed several potential confounders of ABT such as sex, BMI, comorbidities, anticoagulation medication and neoadjuvant chemotherapy, preoperative chest surgery and duration of surgery. Interestingly, anticoagulant therapy did not significantly influence ABT use, indicating an efficient anticoagulation/bridging management.

We consistently found that in lung tumor patients Rhesus negativity was associated with perioperative ABTs, while other blood group properties were not. Moreover, the role of Rhesus negativity depended on the presence or absence of anemia. Few papers have addressed the influence of Rhesus (D) status on perioperative anemia or need for ABTs [18, 19], and a convincing molecular mechanism associating Rhesus (D) status with perioperative anemia has not been demonstrated as yet. Here, experimental lung cancer models and experimental models for blood volume regulation and coagulation could provide explanations. However, one must keep in mind that the Rhesus system does only exist in a small number of animals, making the investigation of Rhesus negativity on bleeding and lung cancer biology in commonly used rodent models futile.

Rhesus negativity has previously found to be linked to various solid malignancies including endometrial, breast, gastric and esophageal cancer [20, 21], as well as an increased risk for lung cancer [10], especially small cell lung cancer [22]. Moreover, a specific erythrocyte-immunological relationship between tumor growth in lung adenocarcinoma and Rhesus factor CE, but not D has been described [23]. This finding might reflect the association between Rhesus factor and squamous cell carcinoma but not adenocarcinoma in our cohort. Since Rhesus factor is an easily available clinical parameter, its relationship with the histological subtype, TNM status and perioperative ABT should be further evaluated in prospective patient cohorts.

Apart from the abovementioned parameters included into the regression model, further clinical parameters were plausibly associated to ABT use. Specifically, TNM classification revealed greater tumors (> T1) and hilar lymph node involvement (N1) to be linked to perioperative ABT, in line with previous studies on other resectable solid tumors [24, 25].

The present study has several limitations. Due to its retrospective nature, data were collected from routine clinical documentation and not prospectively assessed for the specific purpose of this study. A second limitation was the relatively low number of ABT events (n = 60), which prevented regression analyses with more than the 6 parameters included, that were selected bases on their clinical meaningfulness. On the other hand, it seems possible that the inclusion of a greater number of predictors would hamper the use of the results in clinical practice. The predictive role of other variables, especially confounders, was analyzed separately by introducing them into the regression model, but all of them did not significantly influence the use of ABT, suggesting the robustness and validity of the selected parameters. Iron deficiency as well as erythropoietin, Vitamin B6/12 and folic acid serum levels were not systematically collected, thus no conclusions regarding reversible causes of the patient’s anemia can be made, although we believe a high share of those to be microcytic anemia of chronic disease associated with tumor.

Since hemoglobin was the strongest predictor for perioperative ABT, we repeated the analysis in patients with and without anemia and found a plausible result rendering it unlikely that the observed associations were due to potential confounding with anemia. In addition, given the low proportion of patients experiencing postoperative bleeding or significant intraoperative blood loss upon major anatomical lung resections as reported in the literature (1.3–2.1%) [2, 3], we may assume that potential confounders did not significantly affect the main findings of our study, although this cannot be excluded.

Any risk management for perioperative ABT should include a preemptive preoperative and intraoperative blood management. This comprises erythropoiesis stimulation (e.g. via iron derivatives, vitamin B complex, erythropoietin analogues), autotransfusion or intraoperative cell salvage therapy. While the use of intraoperative blood salvage is heavily debated for its potential risk of hematogenic dissemination and thrombosis [26], effects of erythropoiesis stimulation are observed 2–4 weeks later. It is a common view that surgical treatment for tumors should not be delayed due to erythropoiesis stimulation [27] and that cancer treatment should not be modified, as anemia correction can be performed in the time left before surgery. Erythropoiesis stimulation and autotransfusion can induce (thrombo-)inflammation, with potential complications, underlining the need for a critical evaluation of these measures. Research on further preoperative ABT risk factors that are feasible in clinical practice might also lead to innovative treatments to reduce ABT requirements in high-risk patients (Additional file 4: Fig. S2).

## Conclusions

Taken together, the present study identified a panel of preoperative, easily available and clinically meaningful parameters that were associated with the risk of perioperative ABT in patients undergoing primary lung cancer resections. This included the result that the predictive value of single parameters depended on the presence of anemia, as well as the intriguing role of the Rhesus factor. These predictors might help in the implementation of preventive strategies for perioperative ABT and the reduction of orderings of blood products. However, the results of this retrospective single-center study should be confirmed in independent cohorts in order to establish their final usefulness (Additional files 4 and 5: Fig. S2 and S3).

## Supplementary Information

**Additional file 1: Table S1.** Binary logistic regression model predicting postoperative ABT requirements (RBCs) in primary lung cancer patients undergoing major surgical resections.

**Additional file 2: Table S2.** Binary logistic regression model predicting postoperative ABT requirements (RBCs and FFPs) in primary lung cancer patients undergoing major surgical resections.

**Additional file 3: Figure S1.** In non anemic patients, multilobar resection and Rhesus factor negativity were associated with increased odds for postoperative ABT.

**Additional file 4: Figure S2.** In non anemic patients, multilobar resections and Rhesus factor negativity were associated with increased odds for postoperative ABT.

**Additional file 5: Figure S3.** In non anemic patients, multilobar resections and Rhesus factor negativity were associated with increased odds for postoperative ABT.

## Data Availability

The datasets of the current study are available from the corresponding author upon reasonable request.

## Abbreviations

ABT: Allogenic blood transfusion
ALAT: Alanine-aminotransferase
ASAT: Aspartat-aminotransferase
BMI: Body mass index
CEA: Carcinoembryonic antigen
CRP: C-reactive protein
CYFRA 21-1: Cytokeratin-fragment 19
DLCO: Diffusing capacity of the lung for carbon monoxide
eGFR: Estimated glomerular filtration rate
FEV_1_: Forced expiratory volume in one second
FFPs: Fresh frozen plasma products
GGT: Gamma-glutamyl transferase
INR: International normalized ratio
LDH: Lactate dehydrogenase
LMU: Ludwig-Maximilians-University of Munich
MCH: Mean corpuscular hemoglobin
MCHC: Mean corpuscular hemoglobin concentration
MCV: Mean corpuscular volume
NSE: Neuron-specific enolase
PTT: Partial thromboplastin time
RBCs: Allogenic red blood cell units
Rh+/−: Rhesus factor positivity/negativity
VC: Vital capacity
ROC: Receiver operating characteristics

## References

[1] Uramoto H, Shimokawa H, Tanaka F (2014). Postoperative bleeding after surgery in patients with lung cancer. Anticancer Res.

[2] Udelsman BV, Soni M, Madariaga ML, Fintelmann FJ, Best TD, Li SS-Y (2020). Incidence, aetiology and outcomes of major postoperative haemorrhage after pulmonary lobectomy. Eur J Cardiothorac Surg.

[3] Panagopoulos ND, Karakantza M, Koletsis E, Apostolakis E, Sakellaropoulos GC, Filos KS (2008). Influence of blood transfusions and preoperative anemia on long-term survival in patients operated for non-small cell lung cancer. Lung Cancer.

[4] Luan H, Ye F, Wu L, Zhou Y, Jiang J (2014). Perioperative blood transfusion adversely affects prognosis after resection of lung cancer: a systematic review and a meta-analysis. BMC Surg.

[5] Cho S, Park J, Lee M, Lee D, Choi H, Gim G (2021). Blood transfusions may adversely affect survival outcomes of patients with lung cancer: a systematic review and meta-analysis. Transl Lung Cancer Res.

[6] Vamvakas EC, Blajchman MA (2010). Blood still kills: six strategies to further reduce allogeneic blood transfusion-related mortality. Transfus Med Rev.

[7] Langer CJ, Choy H, Glaspy JA, Colowick A (2002). Standards of care for anemia management in oncology: focus on lung carcinoma. Cancer.

[8] Cirasino L, Barosi G, Torre M, Crespi S, Colombo P, Belloni PA (2000). Preoperative predictors of the need for allogeneic blood transfusion in lung cancer surgery. Transfusion.

[9] Barrett-Lee PJ, Bailey NP, O’Brien ME, Wager E (2000). Large-scale UK audit of blood transfusion requirements and anaemia in patients receiving cytotoxic chemotherapy. Br J Cancer.

[10] Urun Y, Utkan G, Cangir AK, Oksuzoglu OB, Ozdemir N, Oztuna DG (2013). Association of ABO blood group and risk of lung cancer in a multicenter study in Turkey. Asian Pac J Cancer Prev.

[11] Travis WD, Brambilla E, Nicholson AG, Yatabe Y, Austin JHM, Beasley MB (2015). The 2015 World Health Organization Classification of lung tumors: impact of genetic, clinical and radiologic advances since the 2004 classification. J Thorac Oncol..

[12] Wang T, Luo L, Huang H, Yu J, Pan C, Cai X (2014). Perioperative blood transfusion is associated with worse clinical outcomes in resected lung cancer. Ann Thorac Surg.

[13] Vespasiani-Gentilucci U, de Vincentis A, Ferrucci L, Bandinelli S, Antonelli Incalzi R, Picardi A (2018). Low alanine aminotransferase levels in the elderly population: frailty, disability, sarcopenia, and reduced survival. J Gerontol A.

[14] Takagi S, Sato S, Oh-hara T, Takami M, Koike S, Mishima Y (2013). Platelets promote tumor growth and metastasis via direct interaction between Aggrus/podoplanin and CLEC-2. PLoS ONE.

[15] Tamim H, Habbal M, Saliba A, Musallam K, Al-Taki M, Hoballah J (2016). Preoperative INR and postoperative major bleeding and mortality: a retrospective cohort study. J Thromb Thrombolysis.

[16] Egenvall M, Mörner M, Martling A, Gunnarsson U (2018). Prediction of outcome after curative surgery for colorectal cancer: preoperative haemoglobin, C-reactive protein and albumin. Colorectal Dis.

[17] Nam J-S, Kim W-J, An S-M, Choi D-K, Chin J-H, Lee E-H, Choi I-C (2019). Age-dependent relationship between preoperative serum aminotransferase and mortality after cardiovascular surgery. Aging (Albany NY).

[18] Ni Y, Ding X-H, Xu Z-J, Zhang Z-F, Zhang Y, Gui B (2021). Association of acute normovolemic hemodilution with decreased length of hospital stay in rhesus-negative patients undergoing major cancer surgeries: a retrospective study. Ann Palliat Med.

[19] Pourafkari L, Baghbani-Oskouei A, Savadi-Oskouei S, Ghaffari S, Parizad R, Tajlil A, Nader ND (2019). Prediction model for significant bleeding in patients with supratherapeutic international normalized ratio after oral administration of warfarin. Clin Drug Investig.

[20] Seebacher V, Polterauer S, Reinthaller A, Koelbl H, Achleitner R, Berger A, Concin N (2018). AB0 blood groups and rhesus factor expression as prognostic parameters in patients with epithelial ovarian cancer—a retrospective multi-centre study. BMC Cancer.

[21] Mayer B, Schraut W, Funke I, Jauch KW, Mempel W, Johnson JP, Schildberg FW (1997). The Rhesus D-negative phenotype is an independent predictor of poor prognosis in curatively (RO) resected gastric cancer patients. Br J Cancer.

[22] Cerny T, Fey MF, Oppliger R, Castiglione M, Nachbur B, Gertsch M (1992). Prevalence of the Rhesus-negative phenotype in Caucasian patients with small-cell lung cancer (SCLC). Int J Cancer.

[23] Schulze AB, Schmidt LH, Baie L, Heitkötter B, Kuemmel A, Mohr M (2018). Rhesus CE expression on patient red blood cells is an independent prognostic factor for adenocarcinoma of the lung. Clin Respir J.

[24] Liu X, Ma M, Huang H, Wang Y (2018). Effect of perioperative blood transfusion on prognosis of patients with gastric cancer: a retrospective analysis of a single center database. BMC Cancer.

[25] Seon DY, Kwak C, Kim HH, Ku JH, Kim HS (2020). Impact of perioperative blood transfusion on oncologic outcomes in patients with nonmetastatic renal cell carcinoma treated with curative nephrectomy: a retrospective analysis of a large, single-institutional cohort. Investig Clin Urol.

[26] Waters JH, Donnenberg AD (2009). Blood salvage and cancer surgery: should we do it?. Transfusion.

[27] Fligor SC, Tsikis ST, Wang S, Ore AS, Allar BG, Whitlock AE (2020). Time to surgery in thoracic cancers and prioritization during COVID-19: a systematic review. J Thorac Dis.

