# Supplementary material for: Preoperative risk factors predict perioperative allogenic blood transfusion in patients undergoing primary lung cancer resections: a retrospective cohort study from a high-volume thoracic surgery center
Source: BMC Surg. 2023 Feb 27;23:44. doi: 10.1186/s12893-023-01924-9 (PMC9972742; doi:10.1186/s12893-023-01924-9)
Supplement: Supplementary file 1 — Additional file 1: Table S1. Binary logistic regression model predicting postoperative ABT requirements (RBCs) in primary lung cancer patients undergoing major surgical resections. [file 12893_2023_1924_MOESM1_ESM.docx]

**Additional Table S1: Binary logistic regression model predicting postoperative ABT requirements (RBCs) in primary lung cancer patients undergoing major surgical resections.**

| **Covariates for postoperative ABT** | **Exp(B) [95% CI]** | **P-value** |
| --- | --- | --- |
| Sex (female) | 2.98 [1.42-6.21] | 0.0038 |
| Preoperative anemia | 24.25 [10.74-54.77] | <0.0001 |
| Multilobar resection | 5.28 [2.24-12.47] | 0.0001 |
| Intraoperative blood loss > 250 mL | 7.32 [3.39-15.81] | <0.0001 |
| ALAT < 17.5 IU/L | 4.15 [1.72-10.04] | 0.0016 |
| Thrombocytes > 293.5 /nL | 2.43 [1.17-5.05] | 0.0168 |
| Rh- (dd phenotype) | 3.08 [1.26-7.55] | 0.0141 |
|  |  |  |

Abbreviations: RBCs = red blood cell units; Exp(B) = Odds ratio, 95% Confidence interval [lower bound-upper bound]; ALAT: alanin-aminotransferase; Rh -: Rhesus factor negativity.
