# Supplementary material for: Preoperative risk factors predict perioperative allogenic blood transfusion in patients undergoing primary lung cancer resections: a retrospective cohort study from a high-volume thoracic surgery center
Source: BMC Surg. 2023 Feb 27;23:44. doi: 10.1186/s12893-023-01924-9 (PMC9972742; doi:10.1186/s12893-023-01924-9)
Supplement: Supplementary file 2 — Additional file 2: Table S2. Binary logistic regression model predicting postoperative ABT requirements (RBCs and FFPs) in primary lung cancer patients undergoing major surgical resections. [file 12893_2023_1924_MOESM2_ESM.docx]

**Additional Table S2: Binary logistic regression model predicting postoperative ABT requirements** **(RBCs and FFPs) in primary lung cancer patients undergoing major surgical resections.**

| **Covariates for postoperative ABT** | **Exp(B) [95% CI]** | **P-value** |
| --- | --- | --- |
| Sex (female) | 2.44 [1.23-4.88] | 0.0112 |
| Preoperative anemia | 18.16 [8.73-37.78] | <0.0001 |
| Multilobar resection | 5.79 [2.50-13.38] | <0.0001 |
| ALAT < 17.5 IU/L | 3.98 [1.73-9.16] | 0.0012 |
| Thrombocytes > 293.5 /nL | 2.04 [1.04-4.02] | 0.0390 |
| Rh- (dd phenotype) | 2.84 [1.23-6.59] | 0.0150 |
|  |  |  |

Abbreviations: RBCs = red blood cell units; FFPs: fresh frozen plasma products; Exp(B) = Odds ratio, 95% Confidence interval [lower bound-upper bound]; ALAT = alanin-aminotransferase; Rh - = Rhesus factor negativity.
