# Supplementary material for: Preoperative risk factors predict perioperative allogenic blood transfusion in patients undergoing primary lung cancer resections: a retrospective cohort study from a high-volume thoracic surgery center
Source: BMC Surg. 2023 Feb 27;23:44. doi: 10.1186/s12893-023-01924-9 (PMC9972742; doi:10.1186/s12893-023-01924-9)
Supplement: Supplementary file 3 — Additional file 3: Figure S1. In non anemic patients, multilobar resection and Rhesus factor negativity were associated with increased odds for postoperative ABT. [file 12893_2023_1924_MOESM3_ESM.pptx]

## Slide 1
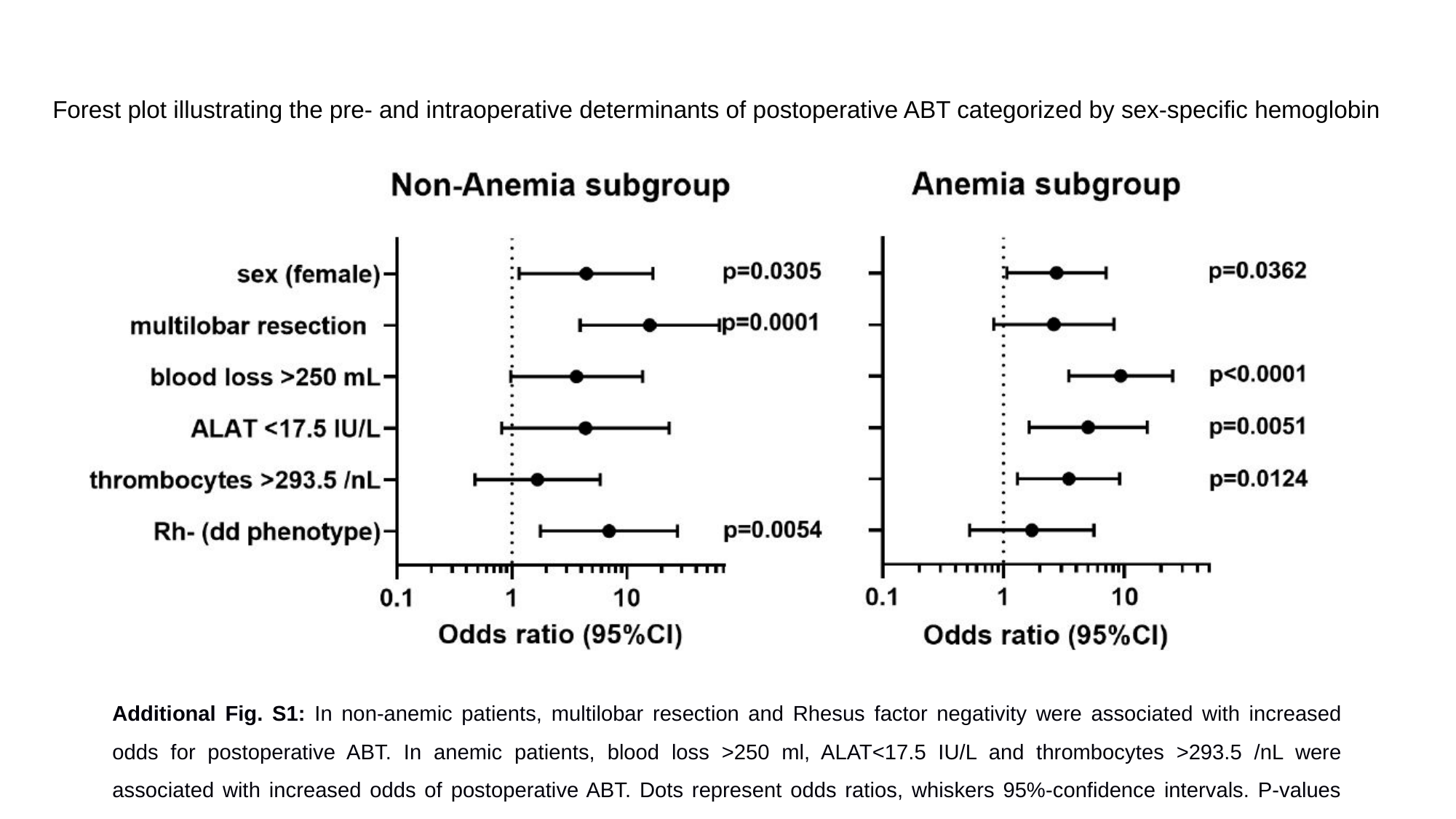

Forest plot illustrating the pre- and intraoperative determinants of postoperative ABT categorized by sex-specific hemoglobin
Additional Fig. S1: In non-anemic patients, multilobar resection and Rhesus factor negativity were associated with increased odds for postoperative ABT. In anemic patients, blood loss >250 ml, ALAT<17.5 IU/L and thrombocytes >293.5 /nL were associated with increased odds of postoperative ABT. Dots represent odds ratios, whiskers 95%-confidence intervals. P-values are also given.
