# Supplementary material for: Preoperative risk factors predict perioperative allogenic blood transfusion in patients undergoing primary lung cancer resections: a retrospective cohort study from a high-volume thoracic surgery center
Source: BMC Surg. 2023 Feb 27;23:44. doi: 10.1186/s12893-023-01924-9 (PMC9972742; doi:10.1186/s12893-023-01924-9)
Supplement: Supplementary file 4 — Additional file 4: Figure S2. In non anemic patients, multilobar resections and Rhesus factor negativity were associated with increased odds for postoperative ABT. [file 12893_2023_1924_MOESM4_ESM.pptx]

## Slide 1
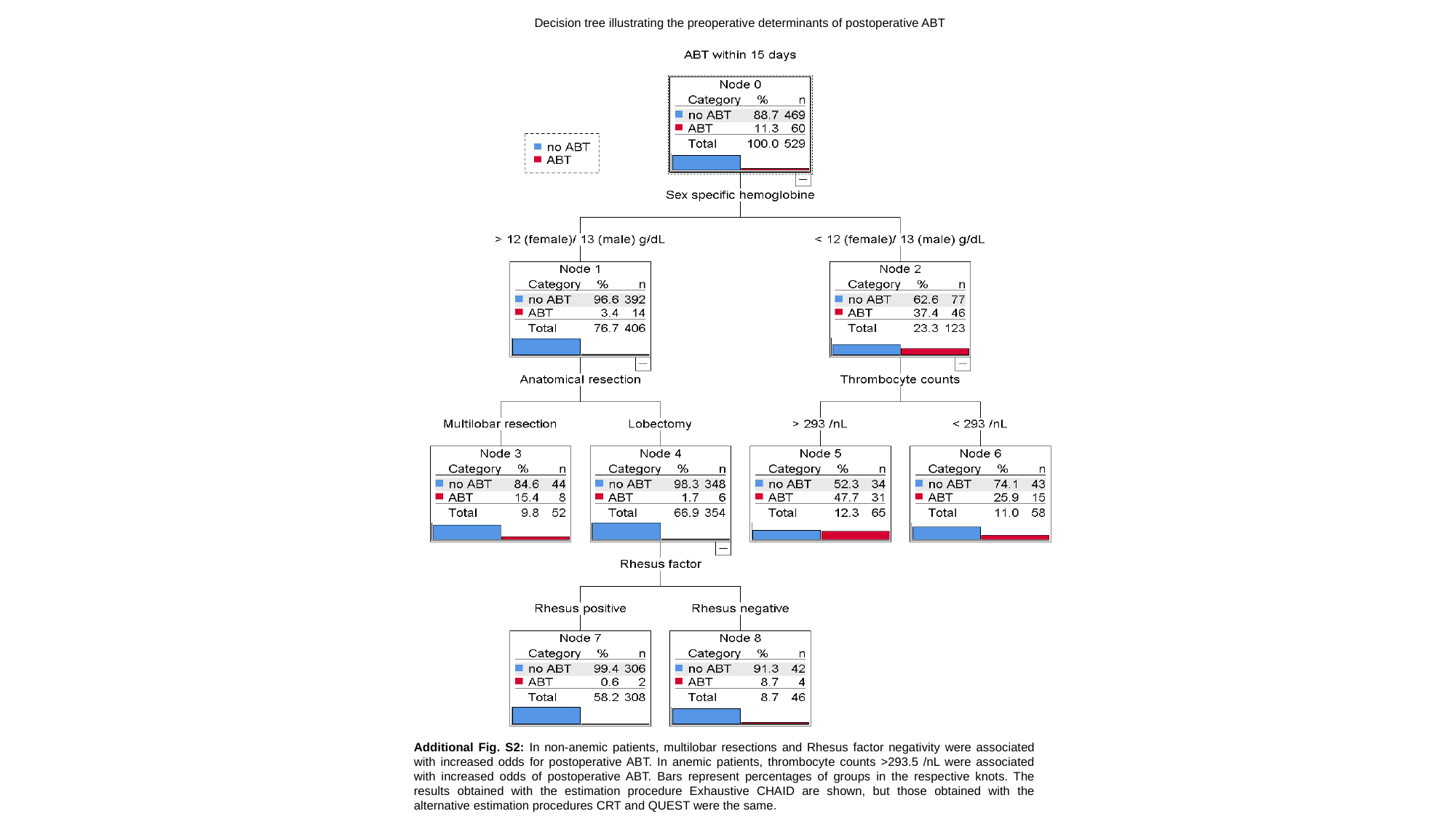

Decision tree illustrating the preoperative determinants of postoperative ABT
Additional Fig. S2: In non-anemic patients, multilobar resections and Rhesus factor negativity were associated with increased odds for postoperative ABT. In anemic patients, thrombocyte counts >293.5 /nL were associated with increased odds of postoperative ABT. Bars represent percentages of groups in the respective knots. The results obtained with the estimation procedure Exhaustive CHAID are shown, but those obtained with the alternative estimation procedures CRT and QUEST were the same.
