# Supplementary material for: Preoperative risk factors predict perioperative allogenic blood transfusion in patients undergoing primary lung cancer resections: a retrospective cohort study from a high-volume thoracic surgery center
Source: BMC Surg. 2023 Feb 27;23:44. doi: 10.1186/s12893-023-01924-9 (PMC9972742; doi:10.1186/s12893-023-01924-9)
Supplement: Supplementary file 5 — Additional file 5: Figure S3. In non anemic patients, multilobar resections and Rhesus factor negativity were associated with increased odds for postoperative ABT. [file 12893_2023_1924_MOESM5_ESM.pptx]

## Slide 1
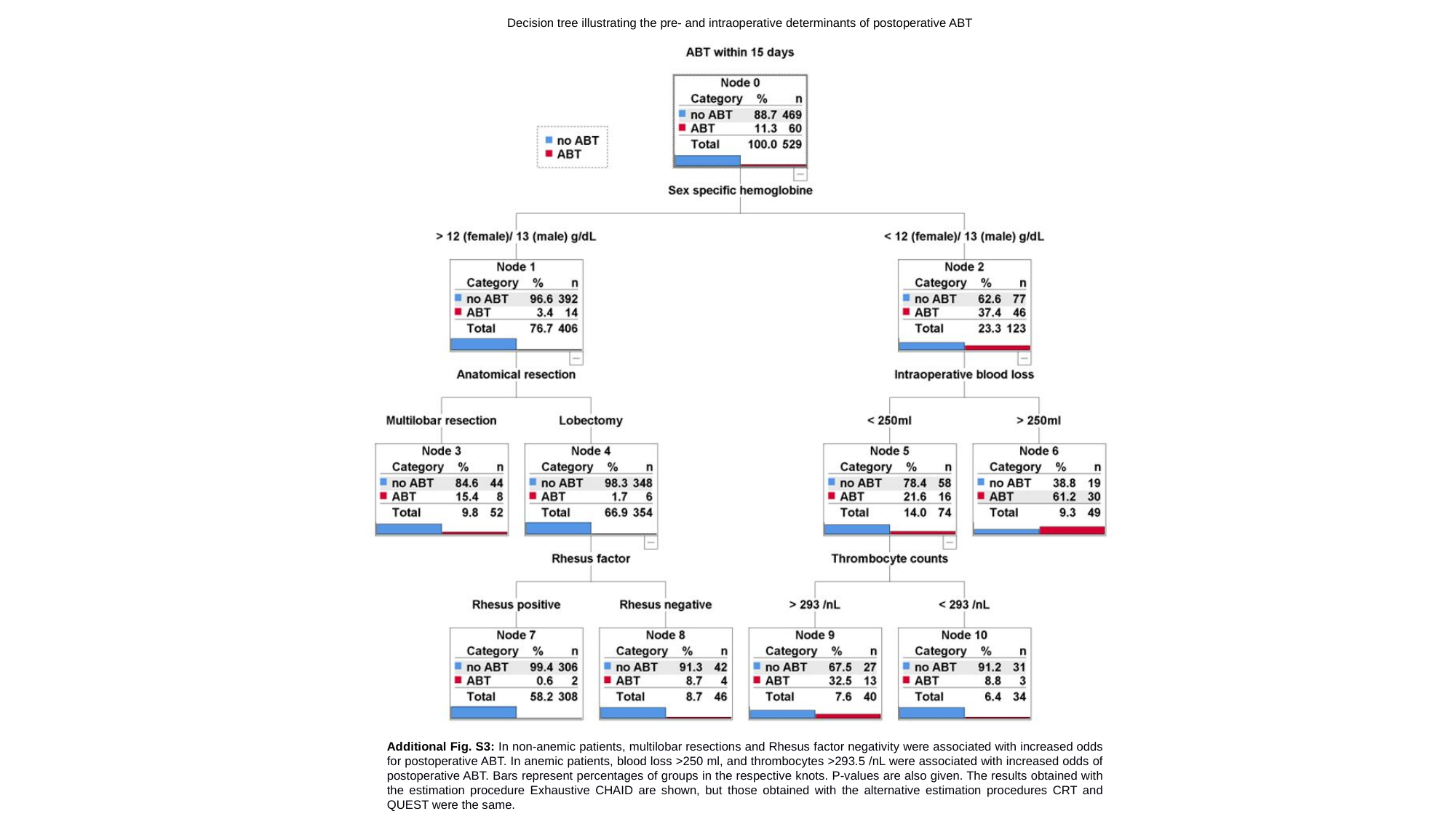

Decision tree illustrating the pre- and intraoperative determinants of postoperative ABT
Additional Fig. S3: In non-anemic patients, multilobar resections and Rhesus factor negativity were associated with increased odds for postoperative ABT. In anemic patients, blood loss >250 ml, and thrombocytes >293.5 /nL were associated with increased odds of postoperative ABT. Bars represent percentages of groups in the respective knots. P-values are also given. The results obtained with the estimation procedure Exhaustive CHAID are shown, but those obtained with the alternative estimation procedures CRT and QUEST were the same.
